# Classification of E-Nose Aroma Data of Four Fruit Types by ABC-Based Neural Network

**DOI:** 10.3390/s16030304

**Published:** 2016-02-27

**Authors:** M. Fatih Adak, Nejat Yumusak

**Affiliations:** Computer Engineering Department, Computer and Information Sciences Faculty, Sakarya University, 2nd Ring Street, Esentepe Campus, Serdivan, Sakarya 54187, Turkey; nyumusak@sakarya.edu.tr

**Keywords:** aroma data, e-nose, ABC, neural networks, sensors

## Abstract

Electronic nose technology is used in many areas, and frequently in the beverage industry for classification and quality-control purposes. In this study, four different aroma data (strawberry, lemon, cherry, and melon) were obtained using a MOSES II electronic nose for the purpose of fruit classification. To improve the performance of the classification, the training phase of the neural network with two hidden layers was optimized using artificial bee colony algorithm (ABC), which is known to be successful in exploration. Test data were given to two different neural networks, each of which were trained separately with backpropagation (BP) and ABC, and average test performances were measured as 60% for the artificial neural network trained with BP and 76.39% for the artificial neural network trained with ABC. Training and test phases were repeated 30 times to obtain these average performance measurements. This level of performance shows that the artificial neural network trained with ABC is successful in classifying aroma data.

## 1. Introduction

Today, electronic nose technology is widely used in various fields, such as food quality, health-care, the defense industry, and environmental studies [1,2,3]. Frequently, odorant samples are measured with an electronic nose, and either the odor data are classified using various algorithms or a certain odor is detected in an odorant mixture. Methods such as Principle Component Analysis (PCA), Hierarchical Cluster Analysis (HCA), and Linear Discriminant Analysis (LDA) are used to decrease the dimension of the odor data, while machine learning and decision support systems are generally used during classification.

Four different fruit aromas (strawberry, lemon, cherry, and melon) were measured in this study with the MOSES II electronic nose, and the relevant aroma data were collected. These four different fruits were selected because they have very different odor characteristics, therefore generalizations can be made [4]. The MOSES II electronic nose has been widely used by other researchers and has produced successful results [5,6,7]. Here, the backpropagation (BP) algorithm and the artificial bee colony algorithm are employed separately for artificial neural network training. The electronic nose test dataset is used in these trained artificial neural networks to measure their performance in novel odorants. Notably, the artificial bee colony-based artificial neural network was found to be highly successful in odor classification. This method can be used to classify fruits and vegetables in wholesale markets to distinguish between specific varieties.

An artificial neural network (ANN) is a computational model that simulates the human brain and its working principles to enable artificial learning [8]. The artificial bee colony (ABC) is a colony optimization algorithm that is based on the working and food searching processes of bees. The algorithm was first proposed by Karaboga, and has since been applied successfully in numerous research studies [9]. In addition to the ANN-ABC method, other similar methods are used by researchers to classify aroma data. In one of these studies, aroma data, which were used in classifying strawberry juice kept in different environments, was trained online by using Extreme Learning Machine (ELM), and successful results were obtained [10]. In another study where strawberry aroma in chewing gums was analyzed, GC-MS was used and a high level of success was achieved [11].

PCA and HCA were used to classify famous Chinese liquor aroma mixtures, where 86 different aroma mixtures were used [12]. Liu *et al.*, however, claimed that analysis methods are not sufficient to achieve highly successful results, and besides PCA and Cluster Analysis (CA), they used BP-ANN and Support vector machine (SVM) algorithms to classify Chinese drinks in eight different tastes for quality and taste assessment [13]. Surprisingly, the same level of success was obtained also in the classification of black tea [14,15,16,17,18,19]. In another study using aroma data, Gupta *et al.* used first PCA and later LDA to classify grape and apple fruits in a chemometric approach, and obtained a 100% success level [20]. Similar to Gupta’s study, PCA and LDA were used to classify polish honey types [21]. Versari *et al.* also employed a chemometric approach to analyze wine quality [22]. In another study using PCA and LDA, the *Orthosiphon stamineus* plant was successfully classified by data fusion [23]. Gas chromatography was employed in another study to detect mango fruit maturity using electronic nose data [24]. Li *et al.* used both artificial neural networks and bayesian networks to detect rotten fruits. They succeeded in decreasing the failure level to 1.8% by using different electronic noses [25].

Artificial neural networks are used extensively in the classification of electronic nose data, and the reader is referred to many other studies on the topic [25,26,27,28,29,30]. However, although ANN give successful results, they also have weaknesses, such as over-fitting of data and getting stuck in local minima. These shortcomings prevent high performance in many data sets. Researchers try to overcome these shortcomings by optimizing training of artificial neural networks. In this study, the ABC algorithm is used to optimize the learning ability of artificial neural networks. Uzlu *et al.* trained artificial neural networks with ABC to predict annual hydroelectric production, and obtained favorable results [31]. Ozkan *et al.* used the same approach to detect oil spillage. Their tests showed that ABC-based training is more successful than BP-based training [32]. Nasimi *et al.* also optimized ANN using ABC, and were 99% successful in predicting bottom hole pressure [33].

Genetic algorithms are one of the other optimization methods used in ANN training. In one study, genetic algorithms were used in copper nanowires loaded on activated carbon, and successful results were obtained [34]. Particle swarm optimization is another frequently used method in ANN optimization [35,36,37,38]. Saraoglu *et al.* used Radial Basis Function ANN to measure blood glucose level from exhaled breath [39], and Gulbag *et al.* used it to identify individual gas concentrations in binary gas mixtures. In both studies, Radial Basis Function ANN produced better results than artificial neural networks with a traditional learning method [40]. In another study that used artificial neural networks in gas mixture quantification, the Hilbert–Huang transform method was used together with an artificial neural network-based pattern recognition method, and an error rate of 7%, 8%, and 12% was achieved [41].

All of the studies discussed here show that ANN alone does not give satisfactory results every time. The incorporation of hybrid studies is necessary to improve the training results. In this study, ANN training is performed separately with the ABC and BP algorithms. A Microsoft Visual C# application is developed to obtain algorithm parameters for inputs, using a few steps. Users can obtain useful comparative results by examining visual graphic displays supplied by the application.

The main goal of this study is the use of the ANN-ABC algorithm to accurately classify electronic nose aroma data. Performance of the algorithm is measured via aroma data, which were never shown before, as the test data.

The rest of the paper is organized as follows: a detailed explanation of the ANN structure and ABC algorithm is provided in the Section 2. Classification of the aroma data are also provided in this section. Test results are given in the Section 3, while conclusions are presented in the Section 4.

## 2. Methodology

### 2.1. Preparation of Data

Aroma data were obtained by measuring four different fruit aromas (melon, strawberry, lemon, and cherry) with the MOSES II electronic nose. The MOSES II electronic nose is being performed at Scientific and Technological Research Council of Turkey (TUBITAK), Marmara Research Center, Materials Institute, Sensor Laboratory directed by Cihat Tasaltin. Test measurements were performed in this laboratory. Temperature and humidity sensors are located in the electronic nose as well. While aromas were presented in 25 mL vials in a headspace sampler to the electronic nose, the sensor temperatures were maintained at 153 ${}^{\circ }$C. Each aroma was measured four times at the same concentration and every time obtained similar values. Every time for each aroma injection took 10 s, and was followed by a 10 min cleaning step using synthetic air.

**Figure 1:**
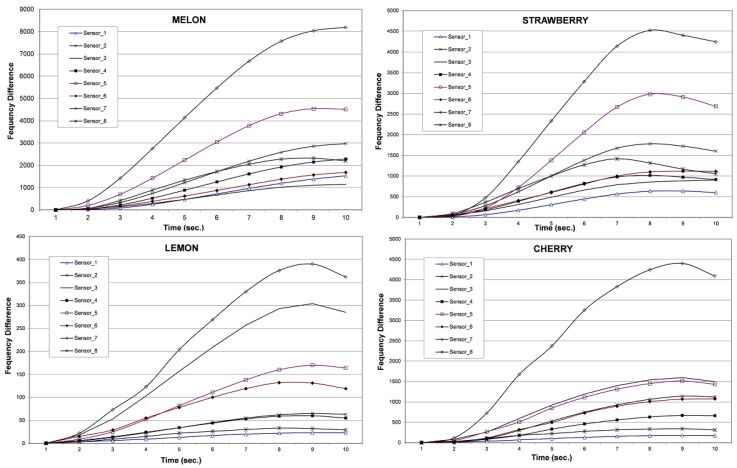
Response of eight sensors to each set of fruit aroma.

The response of each sensor to each set of fruit aroma data is shown in Figure 1. The responses become stronger gradually, reach their maximum values, and either stay there or begin to decrease. Maximum response values were used for the analysis. This maximum value was reached in 10 s for some sensors, while for others it was reached earlier, followed by a decrease in response intensity.

**Figure 2:**
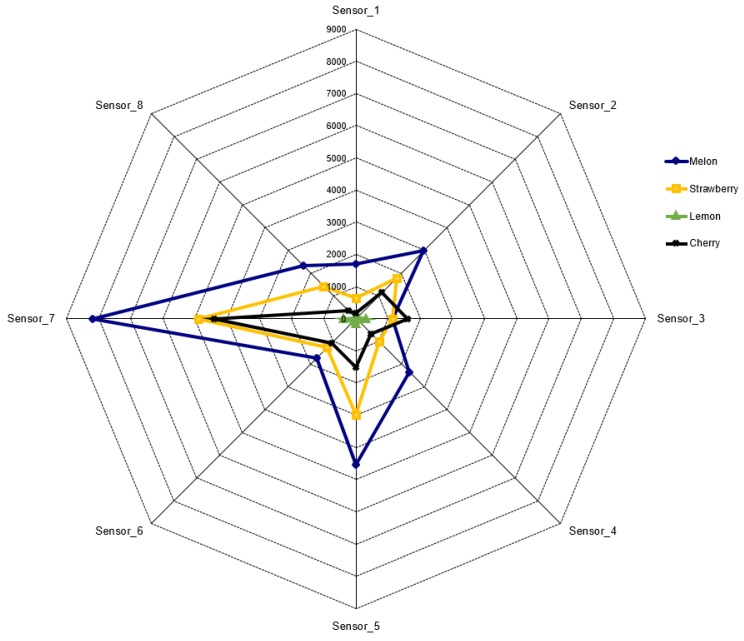
Radar plots for four fruits.

Maximum response values for the eight sensors for each fruit aroma were analyzed using a radar graphic. Observation of the results reveals that each fruit aroma has its own fingerprint, which allows successful distinction of each (Figure 2).

Figure 2 shows that the sensor response values are large numbers. However, in this study, since a sigmoid function was used as the activation function of the ANN, the response value of the sensors should be reduced to [0,1] range. Therefore, column-based min-max normalization was applied to the dataset Equation (1). $x_{i}$ is the value to be normalized, and $x_{\min}$ and $x_{\max}$ are the minimum and maximum values of the dataset. Table 1 shows the normalization of the responses of the first sensor to melon aroma, as an example.

(1) $$x_{i,\;0\;\;to\;1}=\frac{x_{i}-x_{\min}}{x_{\max}-x_{\min}}$$

### 2.2. Artificial Neural Network (ANN)

**Figure 3:**
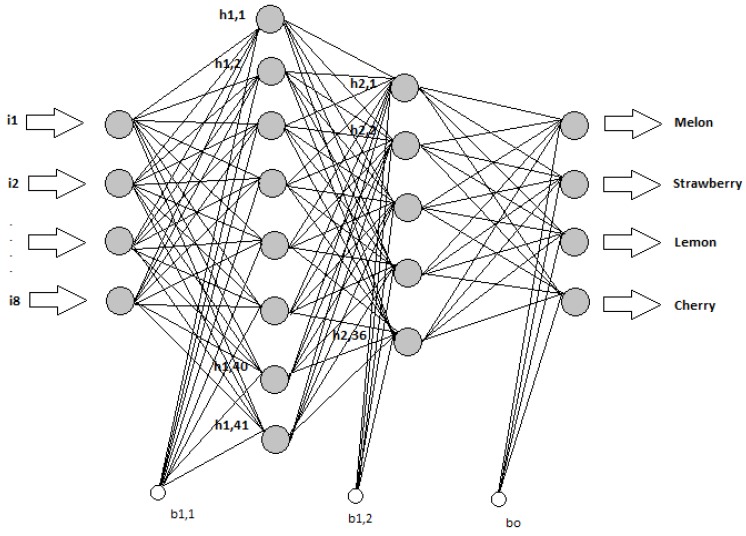
Structure of the artificial neural network (ANN) model used in the study.

An artificial neural network consists of layers of process elements called neurons. A basic ANN model consists of three layers: an input layer, a hidden layer, and an output layer. The number of hidden layers may be increased as much as required. The ANN model designed in this study, shown in Figure 3, achieved the best performance of all the models in the aroma data test.

Figure 3 shows that the ANN model includes one input layer, one output layer, and two hidden layers. The input layer consists of eight inputs, which are sensor responses of the electronic nose. The first and second hidden layers include 41 and 36 neurons, respectively. The output layer has four outputs, each for a distinct fruit. The feed-forward ANN model was then trained using a backpropagation algorithm with the parameters given in Table 2.

The process of presenting one line of the dataset to the network is known as an iteration, and presenting the complete training dataset is termed an epoch in feed-forward neural networks. Net value of a neuron is calculated in Equation (2).

(2) $$NET_{j}^{a}=\sum_{k=1}^{n}x_{k}^{i}w_{kj}$$

where *n* is the total number of inputs applied to neuron *k*. $w_{kj}$ represents weight degree between neuron *k* and *j*. Since the sigmoid activation function is used here, the output value of a neuron is given by Equation (3).

(3) $$O_{j}^{a}=\frac{1}{1+e^{-(NET_{j}^{a}+\beta_{j}^{a})}}$$

where $\beta_{j}^{a}$ is the threshold value for the *j-th* element in the hidden layer. All the hidden and the output layer values are calculated to obtain the output of the network. The difference between the expected output ($T_{m}$) and the obtained output ($O_{m}$) gives the error of the network for that iteration (Equation (4)).

(4) $$E_{m}=T_{m}-O_{m}$$

The backpropagation algorithm aims to minimize the error given by Equation (4). Total error is defined as the sum of squares of all errors in all iterations (Equation (5)).

(5) $$E_{total}=\frac{1}{2}\sum_{m=1}E_{m}^{2}$$

The mse (mean squared error) is calculated by taking the average of the epoch errors (Equation (6)).

(6) $$mse=\frac{1}{n}\sum_{k=1}^{n}E_{total}$$

The backpropagation algorithm uses error rates to update network weights. First, weight changes are calculated, then network weights are updated, and thresholds are updated in the same way (Equations (7)–(11)).

(7) $$\delta_{o}=O_{o}\left(1-O_{o}\right)E_{m}$$

(8) $$\Delta w_{ij}=\lambda \delta_{o}O_{i}+\alpha \Delta w_{ij}\left(t-1\right)$$

(9) $$w_{ij}=w_{ij}\left(t-1\right)+\Delta w_{ij}$$

(10) $$\Delta \beta_{ji}=\lambda \delta_{o}+\alpha \Delta \beta_{ji}\left(t-1\right)$$

(11) $$\beta_{ji}=\beta_{ji}\left(t-1\right)+\Delta \beta_{ji}$$

where $\Delta w_{ij}$ is the weight changing rate and $\delta_{o}$ is the local gradient and $O_{o}$ is the output for neuron *o*. Learning rate, denoted by *λ*, determines the rate of learning in each step. Whereas momentum, denoted by *α*, tries to keep the network out of local minima. In this study, the training dataset selected from the fruit aroma data were presented to the network. The number of epochs used in the ANN was set to 10,000.

### 2.3. Artificial Bee Colony Algorithm (ABC)

The Artificial Bee Colony algorithm is a colony optimization algorithm that simulates the behavior of bees while finding food sources. It was first developed by Karaboga [42]. Employee bee, onlooker bee, and scout are the three types of bees in the algorithm. The nectar of each food source is collected by an employee bee. Scout bees randomly search for new food sources, and they become employee bees once they find a food source. Scout bees share the information about food sources rich in nectar with onlooker bees in the dance area. Communication in the bee colony is established in this way. In the ABC algorithm, food sources are determined randomly in the first step (Equation (12)).

(12) $$x_{ij}=x_{\min j}+rand\left(0,1\right)\left(x_{\max j}-x_{\min j}\right)$$

where *i* represents the food source and *j* represents the parameter to be optimized. The expression rand(0,1) generates a random value between 0 and 1. Employee bees are sent to the food source area, and new food sources are determined in the neighborhood of the existing food source (Equation (13)).

(13) $$v_{ij}=x_{ij}+\varphi_{ij}\left(x_{ij}-x_{kj}\right)$$

where *k* is from food source and *j* from optimization parameters are randomly chosen indexes. $\varphi_{ij}$ is a random number that controls the production of neighbor food sources. The fitness value of the newly found food source is determined by the fitness function given in Equation (14). If the nectar amount of the new source is lower than that of the existing one, food source is not changed, and the bees continue to exploit the existing source.

(14) $$fitness_{i}=\left\{\begin{array}{c} \frac{1}{1+f_{i}}\;\;\;\;\;\;\;\;\;\;\;\;\;\;f_{i}\geq 0\\ \frac{1}{abs(f_{i})}\;\;\;\;\;\;\;\;\;f_{i}<0 \end{array}\right\}$$

where $f_{i}$ is the fitness value of the solution *i*, which has relation with the nectar amount of food source in position *i*. Table 3 gives the parameters of the ABC algorithm, and their values in this study.

The ABC algorithm tries to reach the global minimum of the function to be optimized using the method described above.

### 2.4. Training ANN with ABC Algorithm

**Figure 4:**
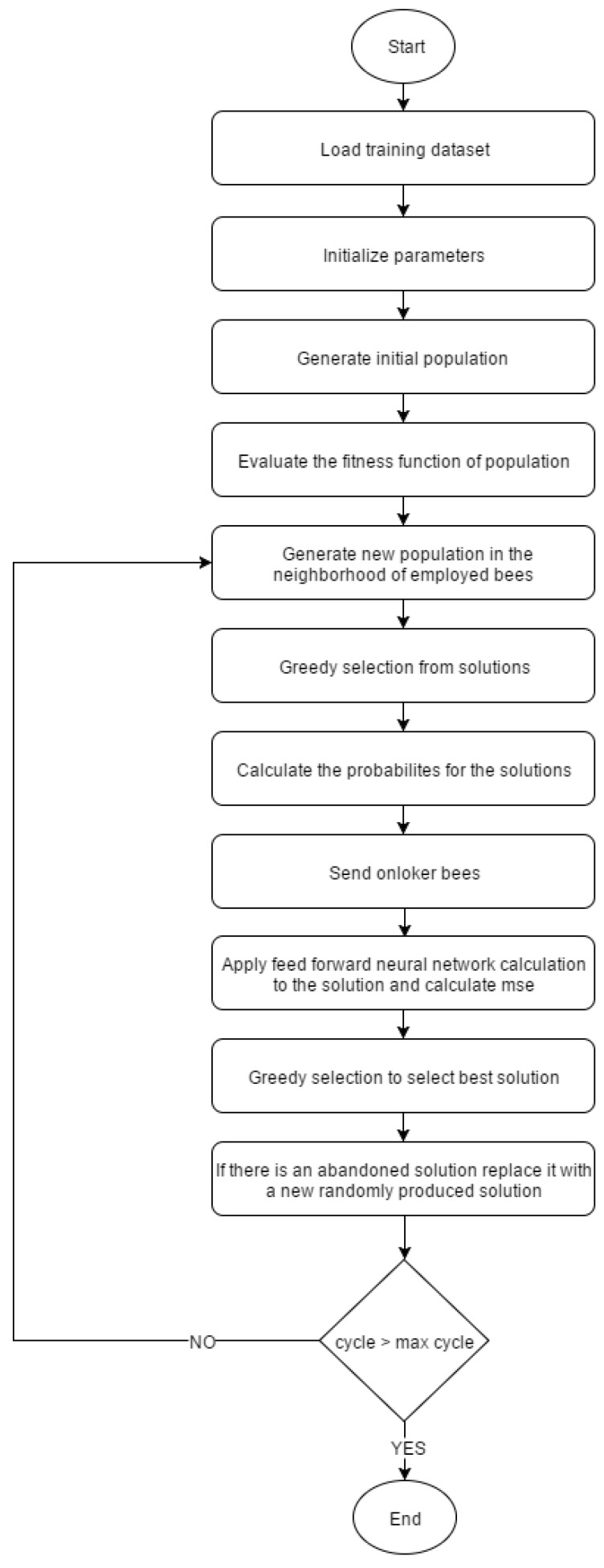
Flowchart of ABC-based ANN.

Although artificial neural networks are widely used in the classification of electronic nose data, they may over-fit the data or get stuck in local minima [8]. Various optimization algorithms are used to resolve these problems of ANN. Here, the ABC algorithm is used as an optimization algorithm in the training of ANN. The ABC algorithm requires only a few parameters to be determined. Therefore, implementation of the algorithm in ANN training is fairly easy. A flowchart of ANN training with the ABC algorithm is given in Figure 4.

Because the network structure given in Figure 3 is used here, 2029 parameters, as calculated in Table 4, are to be optimized by the ABC algorithm. The ABC algorithm tries to find the optimum values of these parameters.

These 2029 parameters were optimized in each cycle of the ABC algorithm, the complete dataset was presented to the network with these parameter values, and the mse value was calculated. This mse value determined the behavior of the algorithm in the next cycle. A general feed-forwarding technique was used in this process of presenting the dataset to the network with new parameter values and calculating the mse value.

Software was developed using Microsoft Visual C# to train the artificial neural network with the ABC algorithm. User input for the algorithm parameters is enabled through an interface design. Once the input parameters are supplied and the software is run, the network design is trained separately with the backpropagation and the ABC algorithm. Part of the dataset is not used in the training phase, and is presented as a test dataset to the trained network. Performance measurements were taken using the same test dataset in both BP-based and ABC-based ANNs. Comparative graphics were supplied in the results window of the interface to enable comparison of the algorithms.

## 3. Results and Discussion

**Figure 5:**
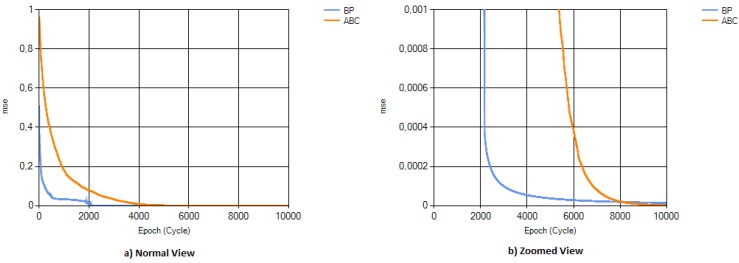
ANN-Backpropagation (BP) and ANN-ABC mean squared error (mse) results.

Aroma data were obtained from eight metal oxide sensors of the MOSES II electronic nose by measuring melon, strawberry, lemon, and cherry aromas. The fruit aroma data were processed, as detailed in Section 2.1, and a dataset of 36 samples was organized. The dataset was separated, using 66.67% as the training set, and the remaining 33.33% as the test dataset. The ANN was trained for 10,000 epochs in BP-based ANN, and for 10,000 cycles for ABC-based ANN. Training and test cases were repeated 30 times. At the end of the 30 runs, mse values obtained for both of the training algorithms were graphed, as shown in Figure 5. It can be observed from the graph that the mse value for the BP algorithm decreases up to 0.0000145, while a much lower value of 0.0000019 is obtained in the ABC case. Moreover, some instability is observed in the mse graph of BP; however, ABC has a smoother decrease pattern in its mse graph.

**Figure 6:**
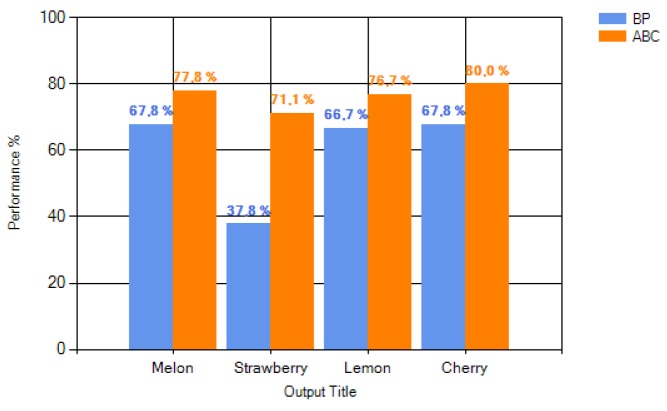
Performances of BP and ABC algorithms on test dataset.

**Figure 7:**
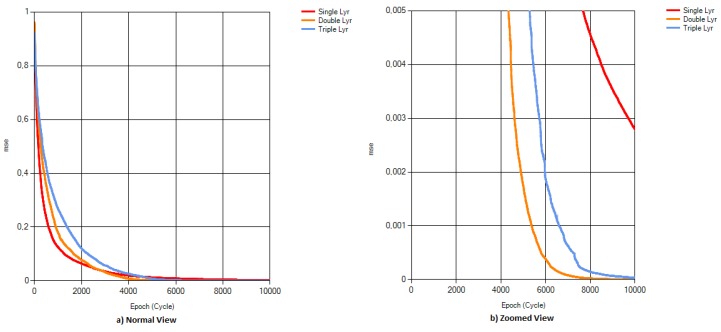
Effect of hidden layer number on mse in the ABC-based ANN model.

A test dataset was presented to ABC-based and BP-based networks with the mse values shown in Figure 5, and the performance of each training algorithm was recorded. Performance results of the networks are given in Figure 6. It can be easily observed that ANN-ABC has a higher performance level for all four of the aroma types. The average performance level of ANN-BP turns out to be much lower than that of the ANN-ABC because of the low performance of ANN-BP (37.8%) for strawberry aroma. ANN-BP has an average performance of 60%, while ANN-ABC has an average performance of 76.39%. To search whether it was possible to find better mse values for ANN-BP, several network designs were used, and the best mse values are listed in Table 5. As can be observed from the table, none of the mse values are lower than the mse value obtained through ANN-ABC. If the number of hidden layers is increased above two, mse (mean squared error) value is increased, and the ANN model does not perform well, as shown by examining the test data. These results reveal that if the optimum ANN model in Figure 3 is extended, the model will over-fit the data. Models with one or three hidden layers have given higher mse values and lower performance results. The effect of hidden layer number on mse in the ABC-based ANN model is shown in Figure 7. Increasing the number of hidden layers up to two results in a decrease in the mse value and higher performance results. When the training is conducted with one hidden layer, the minimum mse value was obtained as 0.0027, while this value decreases down to its lowest value of 0.0000019 with two hidden layers. However, when the number of hidden layers was increased to three, this mse value increased to 0.000031. These trials showed that the lowest mse value was obtained with two hidden layers.

## 4. Conclusions

The software developed in this study enables training of a network both with BP and ABC algorithms. The network is designed by the user, using the software according to the input dataset. The parameters required by the algorithms are supplied as inputs by the user through an interface. Users can easily compare performances of the algorithms using graphical user interfaces of the software. In this study, the dataset was constructed by measuring four different fruit aromas with the MOSES II electronic nose and normalizing the data. To overcome over-fitting and becoming stuck in local minima weaknesses of artificial neural networks, training of the ANN was optimized with the ABC algorithm. As an optimization algorithm, ABC requires only a few parameters, which makes it fairly easy to implement. This process is also successful in obtaining the global minimum. The network designed here consists of four layers, requires eight inputs, and produces four outputs. The training and testing of this network were repeated 30 times. ANN-BP achieved a performance of 60% in these tests, while ANN-ABC reached 76.39%. In particular, the low performance (37.8%) of ANN-BP in the strawberry aroma was not observed for ANN-ABC (71.1%). The results showed that training the artificial neural network with the ABC optimization algorithm provides successful results in classification of electronic nose data.

## Figures and Tables

**Table 1:** Response of sensor 1 to melon aroma.

Seconds	Frequency Difference								Norm
1	0	0	0	0	0	0	0	**0**	**0.0000**
2	0	0	0	0	0	0	0	**14**	**0.0091**
3	0	0	0	0	0	0	14	**89**	**0.0581**
4	0	0	0	0	0	14	89	**251**	**0.1639**
5	0	0	0	0	14	89	251	**472**	**0.3083**
6	0	0	0	14	89	251	472	**718**	**0.4690**
7	0	0	14	89	251	472	718	**965**	**0.6303**
8	0	14	89	251	472	718	965	**1194**	**0.7799**
9	14	89	251	472	718	965	1194	**1385**	**0.9046**
10	89	251	472	718	965	1194	1385	**1531**	**1.0000**

**Table 2:** Artificial Neural Network parameters.

Parameter	Value
Weight range	[−1, 1]
Threshold range	[−1, 1]
Activation function	Sigmoid
Learning coefficient	0.2
Momentum	0.8
Stopping rule (epoch)	10000

**Table 3:** Artificial bee colony (ABC) algorithm parameters.

Parameter	Value
Parameter’s lower bound	−10
Parameter’s upper bound	10
Colony size	200
Food source limit	1000
Max cycle	10,000

**Table 4:** Number of parameters optimized by the ABC algorithm.

	Number of Parameters
Between input layer and the first hidden layer	8 × 41 = 328
Between the first and the second hidden layer	41 × 36 = 1476
Between the second hidden layer and the output layer	36 × 4 = 144
Number of threshold values in hidden layers and the output layer	41 + 36 + 4 = 81
Total number of parameters	328 + 1476 + 144 + 81 = 2029

**Table 5:** Different attempts to obtain better results in ANN-BP.

Number of Hidden Layers	Number of Neurons	Min mse Value
1	41	0.00023
1	45	0.00101
2	25 + 10	0.0011
2	41 + 36	0.000032
3	41 + 36 + 15	0.0034
